# A nonsense mutation in mouse *Adamtsl2* causes uterine hypoplasia and an irregular estrous cycle

**DOI:** 10.1007/s00335-023-10016-1

**Published:** 2023-09-01

**Authors:** Yuka Iwanaga, Kaori Tsuji, Ayaka Nishimura, Kouji Tateishi, Misa Kakiuchi, Takehito Tsuji

**Affiliations:** Graduate School of Environmental, Life, Natural Science and Technology, Okayama University, Okayama, 700-8530 Japan

## Abstract

The spontaneous mutation stubby (*stb*) in mice causes chondrodysplasia and male infertility due to impotence through autosomal recessive inheritance. In this study, we conducted linkage analysis to localize the *stb* locus within a 1.6 Mb region on mouse chromosome 2 and identified a nonsense mutation in *Adamtsl2* of *stb*/*stb* mice. Histological analysis revealed disturbed endochondral ossification with a reduced hypertrophic chondrocyte layer and stiff skin with a thickened dermal layer. These phenotypes are similar to those observed in humans and mice with *ADAMTSL2/Adamtsl2* mutations. Moreover, *stb*/*stb* female mice exhibited severe uterine hypoplasia at 5 weeks of age and irregular estrous cycles at 10 weeks of age. In normal mice, *Adamtsl2* was more highly expressed in the ovary and pituitary gland than in the uterus, and this expression was decreased in *stb*/*stb* mice. These findings suggest that *Adamtsl2* may function in these organs rather than in the uterus. Thus, we analyzed *Gh* expression in the pituitary gland and plasma estradiol and IGF1 levels, which are required for the development of the female reproductive tract. There was no significant difference in *Gh* expression and estradiol levels, whereas IGF1 levels in *stb*/*stb* mice were significantly reduced to 54–59% of those in +/+ mice. We conclude that *Adamtsl2* is required for the development of the uterus and regulation of the estrous cycle in female mice, and decreased IGF1 may be related to these abnormalities.

## Introduction

Mice homozygous for the spontaneous mutation stubby (*stb*) exhibit chondrodysplasia characterized by mild growth retardation and disproportionate dwarfism (Miller and Flynn-Mille 1976). Mice with the *stb*/*stb* genotype are distinguishable from normal (+/?) mice because of smaller body sizes starting from approximately 1 week postpartum. From 20 to 27 days of age, the body length of *stb*/*stb* mice is approximately 86% of that of normal mice. The body weights of *stb*/*stb* mice are 89% of those of normal mice, and the weights of the spleen, thymus, and heart are reduced by 77–92%. The largest differences in weight are observed in the testes, which develop to only 57% of the testicular weight of normal mice from 29 to 32 days of age. At 60 days of age, the difference from normal mice improves to 73%, although testicular hypoplasia remains the most serious abnormality after maturation (Lane and Dickie 1968). Furthermore, *stb*/*stb* male mice are infertile despite normal spermatogenesis and testicular steroidogenesis (Chubb and Nolan 1985; Chubb 1987). Intriguingly, a sexual behavior test showed that *stb*/*stb* mice lack intromission and ejaculation, indicating that their infertility is caused by impotence (Chubb and Henry 1987). Thus, *stb*/*stb* mice provide the first animal model for the study of impotence caused by an autosomal gene mutation. In contrast, *stb*/*stb* female mice can reproduce, and the effects of the *stb* locus on the female reproductive system have not been investigated.

The *stb* locus maps between 9.7 cM from the *Etn2* (also called *Sd*) locus and 41.1 cM from the nonagouti locus on chromosome 2 (Lane and Dickie 1968), but this large region prevents efforts to identify the causative gene. The identification of a causative gene within the *stb* locus will contribute insight into chondrodysplasia as well as sexual behavior directly related to male infertility.

In approaching the identification of the causative gene, our research led us to consider the members of the disintegrin-like and metalloproteinase domain with thrombospondin type 1 motifs (ADAMTS) superfamily, which comprises 19 secreted ADAMTS proteases and 7 secreted ADAMTS-like (ADAMTSL) glycoproteins in mammals. ADAMTS proteases possess an N-terminal protease domain containing a catalytic module and a C-terminal ancillary domain containing different modules that determine substrate specificity (Apte 2009; Dubail and Apte 2015). ADAMTS proteases are involved in organ development and tissue homeostasis by regulating extracellular matrix formation, remodeling and homeostatic adaptation (Rose et al. 2021). Compared to the domain structure of ADAMTS proteases, ADAMTSL isoforms commonly lack a protease domain and likely have unique functions through modulating microfibril assembly and signaling networks in the extracellular environment (Apte 2009; Hubmacher and Apte 2015, Mead and Apte 2018). Among the genes encoding the seven ADAMTSLs, *ADAMTSL2* is implicated in the recessive genetic disorder geleophysic dysplasia-1 (GD), which is characterized by severe short stature, cardiac valvular anomalies and skin thickening (Le Goff et al. 2008; Allali et al. 2011). *ADAMTSL4* is the causative gene of ectopia lentis et pupillae, which is characterized by displacement of the lenses and pupils (Ahram et al. 2009). Therefore, ADAMTSLs contribute to the regulation of the extracellular matrix environment, although further studies on the detailed mechanisms of their functions are needed.

In this study, we performed linkage mapping and exome sequence analysis to identify the gene responsible for the *stb* locus and discovered a nonsense mutation in *Adamtsl2*. Furthermore, phenotypic analysis of *stb*/*stb* female mice revealed a previously unidentified physiological role of *Adamtsl2* in female reproductive function.

## Materials and methods

### Mice

Mouse strain heterozygous for *stubby* (*stb*/+) were obtained from the Jackson Laboratory, and the strain was maintained by sib mating of heterozygotes under conditions of 12–12 h light–dark and temperature at 23 °C. C57BL/6, C3H/He and BALB/c mice were purchased from CLEA Japan, Inc. (Tokyo, Japan). All animal experiments were approved by the Animal Committee of Okayama University and were conducted in accordance with the Guidelines for Animal Experiments at Okayama University. The mouse organs and blood were collected after euthanasia by carbon dioxide inhalation.

### Linkage analysis

F_1_ mice were generated by mating heterozygous (*stb*/+) mice with C57BL/6 mice, and 88 affected (*stb*/*stb*) progenies of 405 F_2_ mice obtained from intercrossing heterozygous (*stb*/+) F_1_ mice were used for linkage analysis. Genomic DNA was extracted from mouse livers by phenol/chloroform extraction. PCR reactions for microsatellite and SNP markers proceeded as follows: 35 cycles at 94 °C for 30 s, 55–60 °C for 30 s, and 72 °C for 45 s. The primer sequences of the markers were designed from genomic sequences on mouse chromosome 2 provided by Mouse Genome Informatics (http://www.informatics.jax.org/). The PCR products for microsatellite and SNP markers were analyzed to determine the genotype by agarose gel electrophoresis and sequence analysis, respectively.

### Exome sequencing analysis

Genomic DNA extracted from the liver of *stb*/*stb* and +/+ mice were used to prepare a paired-end library using SureSelectXT Library Prep Kit (Agilent, Japan). Sequencing was performed on Illumina Novaseq 6000 with 150 bp paired-end reads. CLC Genomics Workbench software 21.0.5 was used for trimming, mapping to the mouse reference genome and identifying variants such as the single nucleotide variants, insertions/deletions and splice-site variants.

To detect the mutation in the genomic DNA of mice, PCR fragments, including exon 15 of *Adamtsl2*, were amplified from genomic DNA and analyzed by Sanger sequencing or by restriction enzyme digestion. PCR was performed using mismatch primer (5′-CTGGGGTGGTAGCCTGTTCCTGAG-3′ and 5′-ACCGGTCCCCAGTCCGAGAGAGT-3′) under the following conditions: 94 °C for 2 min followed by 35 cycles of 94 °C for 30 s, 65 °C for 20 s, and 72 °C for 25 s. Amplified fragments were reacted with *HinfI* and the fragment size was checked by 3.5% agarose gel electrophoresis.

### Skeletal preparation and histological analysis

After the skin and internal organs of the mice were removed, the skeletons were fixed in 95% ethanol for 1 day and then stained with 0.15% alcian blue in 80% ethanol and 20% acetic acid for 1 day. Fixed skeletons were dehydrated in 100% ethanol and immersed in 2% KOH for 1–7 days. The skeletons were then stained with 0.015% alizarin red in 1% KOH for 1 day, cleaned in a series of graded glycerin and stocked in glycerin and ethanol 1:1. For histological examination, tibiae, skins and ovaries of *stb*/*stb* and +/+ mice were fixed overnight in Bouin’s solution. Tibias were decalcified in 10% EDTA for 4 days. Specimens were dehydrated in ethanol, embedded in paraffin, sectioned at 4 µm, and stained with hematoxylin and eosin. The images were captured with a DS-Fi3 camera (Nikon, JAPAN). The thicknesses in tibial growth plates and skins were measured at randomly selected three locations in each mouse using NIS-Elements software (Nikon, JAPAN).

### Examination of litter size and estrous cycles

Female mice (*stb*/*stb* and *stb*/+) at 11–12 weeks of age were mated with *stb*/+ male mice. The number of pups at first parturition was counted. Vaginal smears of *stb*/*stb* and +/+ mice were daily at 10 am for 14 days starting at 10 weeks of age. The smears were stained with Giemsa stain solution and define the stage of the estrous cycle as followed; proestrus (predominance of nucleated epithelial cells), estrus (predominance of cornified epithelial cells), metestrus (predominance of cornified epithelial cells and leukocytes) and diestrus (predominance of leukocytes). When proestrus, estrus, metestrus and diestrus were observed in the order within 4–5 days, it was counted as normal estrous cycle.

### Quantitative real-time PCR

Total RNA was extracted using TRIzol Reagent (Thermo Fisher Scientific, Japan). First-strand cDNA was synthesized from total RNA treated with DNase I using PrimeScript II 1st strand cDNA Synthesis Kit (Takara Bio, Japan). Real-time PCR was performed with LightCycler 480 Real-Time PCR System (Roche Diagnostics, Germany) using THUNDERBIRD Probe qPCR Mix (Toyobo, Japan) with Universal Probe Library (Roche Diagnostics, Germany) or TB Green Premix Ex Taq II (Takara Bio, Japan). Duplicate wells were used for each sample, and all reactions were performed in triplicate. Primer sequences and selected probes were as follows: *Adamtsl2*: (5′-gccactgccttcaacagag-3′ and 5′-ctggtaccgtttggacgtg-3′; probe number 98); *Ubb*: (5′- ccaggataaagagggcatcc-3′ and 5′-cagggttgactccttctgga-3′; probe number 110). Predesigned primer and probe were used for *18S rRNA* (PrimerDesign, UK). *Ubb* and *18S rRNA* were used as a reference for normalization. Primer sequences used with an intercalating dye were as follows: *Adamtsl2*: (5′-cacctatgccatgtgtgttcg-3′ and 5′- cgcacgatgcggaactgatg-3′); *Gh*: (5′-agtcctgtggacagatcactg-3′ and 5′-gctcggagcacagcattag-3′); *Gapdh*: (5′-tgatgggtgtgaaccacgag-3′ and 5′-gcccttccacaatgccaaag-3′). Relative quantification was performed using the 2^−∆∆Ct^ method.

### Measurement of serum hormones

For measurement of estradiol and IGF1, plasma was prepared from blood collected from female *stb*/*stb* and +/+ mice at 5 and 10 weeks of age. *stb*/*stb* and +/+ mice at 10 weeks of age were used at the proestrus stage determined by the vaginal smear test. The concentrations of Estradiol and IGF1 were estimated using Estradiol ELISA kit (Cayman Chemical) and IGF1 ELISA (AssayPro).

### Statistical analyses

Data were analyzed using a two-tailed unpaired Student’s *t*-test. A* p* value less than 0.05 was considered statistically significant.

## Results

### Linkage analysis of the *stb* locus

**Fig. 1:**
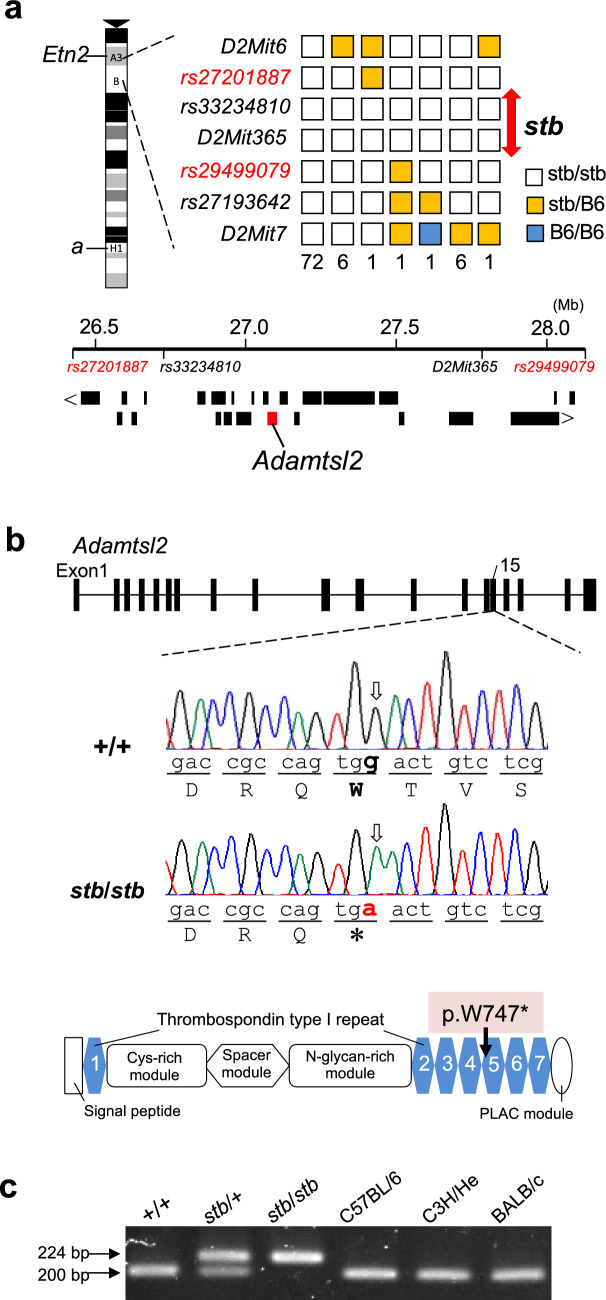
Chromosomal location and identification of the causative mutation of the *stb* locus. **a** Segregation pattern of the *stb* locus and flanking DNA markers in 88 homozygous (*stb*/*stb*) mice of F_2_ progeny by linkage analysis. Each box indicates the genotype of each DNA marker in the F_2_ progeny. The number of mice with each haplotype is given at the bottom of each column. The positions of the genes within the region between *rs27201887* and *rs29499079*, where the *stb* locus was mapped on mouse chromosome 2, are shown below. Each black square line indicates the position of a gene. **b** Nonsense mutation identified in *Adamtls2* of *stb*/*stb* mice. A single nucleotide substitution of G to A and a termination codon introduced within the fifth thrombospondin type 1 motif (p.W747*) was shown in the sequence traces obtained from PCR products amplified from genomic DNA and the structure of ADAMTS2, respectively. **c** Detection of the nonsense mutation in *Adamtls2* by PCR-RFLP analysis. A 224-bp fragment, including the nucleotide substitution of *Adamtls2* of *stb*/*stb* mice, was not digested by the *HinfI* restriction enzyme, while the fragments of +/+ and other normal mice were digested, yielding 200-bp fragments

The *stb* locus resides within the 135 Mb region between the early transposon element insertion site 2 (*Etn2*) and the nonagouti (*a*) loci on chromosome 2, according to Mouse Genome Informatics (http://www.informatics.jax.org). We conducted linkage analysis to more precisely localize the *stb* locus, followed by exome sequencing to identify the causative mutation. To perform linkage analysis, F_2_ mice were generated by mating F_1_ progeny from a cross between *stb* heterozygous mice (*stb*/+) and C57BL/6 mice. The resultant 405 F_2_ offspring included 88 (49 females and 39 males) (*stb*/*stb*) with a dwarf phenotype and 317 (154 females and 163 males) with a normal phenotype (+/?). The ratio of affected to normal F_2_ progeny did not significantly differ from the 1:3 distribution expected for recessive inheritance. We used microsatellite DNA and SNP markers, which are located between the *Etn2* and *a* loci on chromosome 2, to genotype the 88 affected mice. This analysis identified *rs33234810* and *D2Mit365*, which are homozygous for the *stb* mouse allele. This indicates that there was no recombination within the *stb* locus, whereas other markers underwent at least one recombinational event. Thus, the *stb* locus was mapped to an approximately 1.6 Mb region between *rs27201887* (Chr. 2: 26324415 based on GRCm39) and *rs29499079* (Chr. 2: 27951885), where 28 protein-coding genes, including *Adamtsl2,* reside (Fig. 1a).

### Exome sequence analysis of *stb/stb* mice

We next performed whole exome sequence analysis of the genomic DNA of *stb*/*stb* and +/+ mice to identify a specific mutated gene in *stb*/*stb* mice. We obtained approximately 7 Gb of sequence data with a Q30 of 94% from both mouse DNA samples. The data were then mapped to the mouse genome reference sequence (GRCm39), and the average coverage of *stb*/*stb* mice and that of +/+ mice were 42.6 × and 39.8 × , respectively. Comparison with the mouse reference sequence showed that four and three variants were detected within the region from *rs27201887* to *rs29499079* comprising the *stb* locus in *stb*/*stb* and +/+ mice, respectively. These variants each harbored a single nucleotide substitution. Three variants (Chr. 2: 26878102, 26878106, and 26952187) that were detected in *stb*/*stb* and +/+ mice were registered SNPs and were therefore considered polymorphisms in the mouse strain. One variant specifically detected in *stb*/*stb* mice was a nonsense mutation with a G to A substitution (c.2241G > A) in exon 15 of *Adamtsl2* (Fig. 1b). Sanger sequencing of this mutation confirmed that *stb*/*stb* and +/+ mice were homozygous for A and G, respectively. The nonsense mutation introduced a premature termination codon within the tryptophan codon at amino acid residue position 747 within the fifth thrombospondin type 1 motif (Fig. 1b). PCR–RFLP analysis specifically identified a 224-bp fragment containing the nonsense mutation in *stb*/*stb* and *stb*/+ stubby mice. This nonsense mutations were not detected in +/+ stubby mice and in inbred mouse strains (Fig. 1c). Thus, we concluded that the *stb* mutant is a nonsense mutation (c.2241G > A, p.W747*) in *Adamtsl2*.

### Analysis of the longitudinal bone growth and skin of *stb/stb* mice

**Fig. 2:**
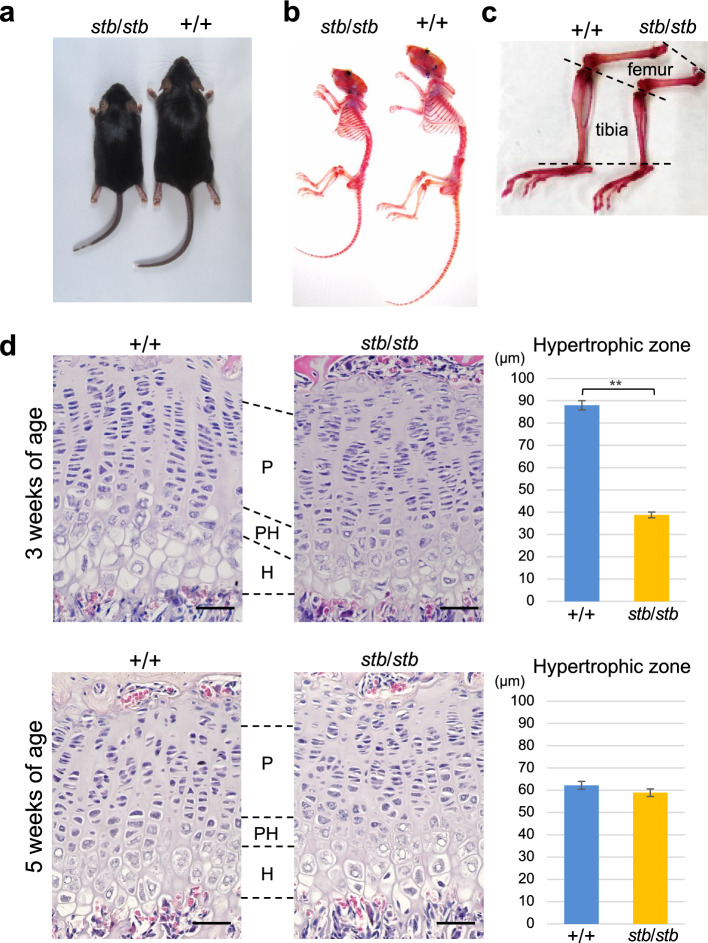
Skeletal morphology and histological analysis of the tibial growth plate in *stb*/*stb* mice. Overall appearance (**a)**, skeletal appearance of the whole structures (**b**) and hind limbs (**c**) of *stb*/*stb* and +/+ mice at 10 weeks of age. **d** Representative sections stained with hematoxylin and eosin of tibial epiphyseal growth plates and thickness of hypertrophic chondrocyte layer of *stb*/*stb* and +/+ mice at 3 weeks (*stb*/*stb* n = 4, +/+ n = 4) and 5 weeks (*stb*/*stb* n = 3, +/+ n = 3) of age. *P* proliferative chondrocyte layer; *PH* prehypertrophic chondrocyte layer; *H* hypertrophic chondrocyte layer. The scale bar in each panel indicates 50 µm. Data are presented as the mean ± SEM. **p < 0.01 indicates differences between *stb*/*stb* and +/+ mice

Short stature due to growth retardation of the long tubular bone resulting from *ADAMTSL2*/*Adamtsl2* mutations is observed in humans (Le Goff et al. 2008), dogs (Packer et al. 2017), and mice (Hubmacher et al. 2015). Mice aged approximately 1 week with the *stb*/*stb* genotype are recognized as those with small bodies, showing obvious dwarfism as they mature (Fig. 2a). We compared the skeletons, which were stained with alcian blue and alizarin red, of *stb*/*stb* mice aged 10 weeks with those of their normal (+/+) littermates. The staining patterns of cartilage and calcified bones of *stb*/*stb* and +/+ mice showed no obvious differences, and the positions and numbers of the skeletal elements of *stb*/*stb* mice were normal (Fig. 2b). However, shortening of the long bones of the femur and tibia was evident (Fig. 2c).

Normal longitudinal bone growth requires sequential and synchronous chondrocyte differentiation through resting, proliferative, prehypertrophic and hypertrophic stages in the growth plate. To identify the basis of the retarded bone growth in *stb*/*stb* mice, we conducted histological analysis of the proximal tibial growth plates. In the tibial growth plate at 3 weeks of age, three to four layers of hypertrophic chondrocytes were observed in +/+ mice. In contrast, one to two layers were observed in the tibial growth plate of *stb*/*stb* mice, and the thickness of the hypertrophic chondrocyte layer was significantly reduced (Fig. 2d). The proliferating chondrocyte layer did not exhibit obvious disorganization in the columnar arrangement, although slightly narrower spacing between proliferating chondrocytes was observed in *stb*/*stb* mice compared with +/+ mice. At 5 weeks of age, the growth plates of *stb*/*stb* and +/+ mice did not exhibit an obvious difference in the layers of hypertrophic chondrocytes (Fig. 2d).

**Fig. 3:**
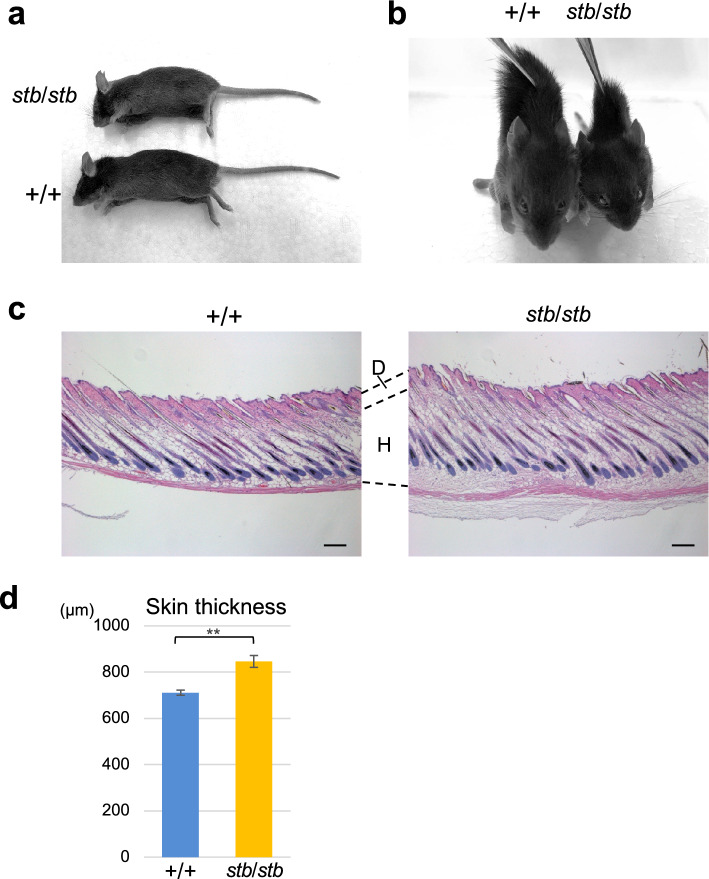
Stiff skin of *stb/stb* mice. **a** Overall appearance of +/+ and *stb*/*stb* littermates at 5 weeks of age. **b** Comparison of skin elongation of *stb*/*stb* and +/+ mice at 5 weeks of age. The skin was pinched with tweezers under anesthesia, and the body was lifted to the same height from the floor. Less elastic skin was observed in *stb*/*stb* mice than in +/+ mice. **c** Representative sections stained with hematoxylin and eosin of the shaved skin on the back of *stb*/*stb* and +/+ littermates at 5 weeks of age. *D* dermis; *H* hypodermis. The scale bar in each panel indicates 100 µm. **d** Skin thickness of *stb*/*stb* (n = 5) and +/+ (n = 5) mice at 5 weeks of age. The skin thickness was measured between the epidermis, dermis and hypodermis. Data are presented as the mean ± SEM. **p < 0.01 indicates differences between *stb*/*stb* and +/+ mice

Next, we analyzed whether the skin of *stb*/*stb* mice was stiff, which is characteristic of human GD and canine Musladin–Lueke syndrome. Obvious changes in appearance were not observed in the skin of *stb*/*stb* mice compared with that of the +/+ mice (Fig. 3a). However, the skin of *stb*/*stb* mice was clearly thicker to the touch and less elastic than that of normal mice (Fig. 3b). Histological analysis revealed that *stb*/*stb* mice had a more noticeable increase in hypodermis, resulting in a significant increase in skin thickness compared with +/+ mice (Fig. 3c and d).

### Analysis of the reproductive tract and estrous cycle of *stb/stb* mice

**Fig. 4:**
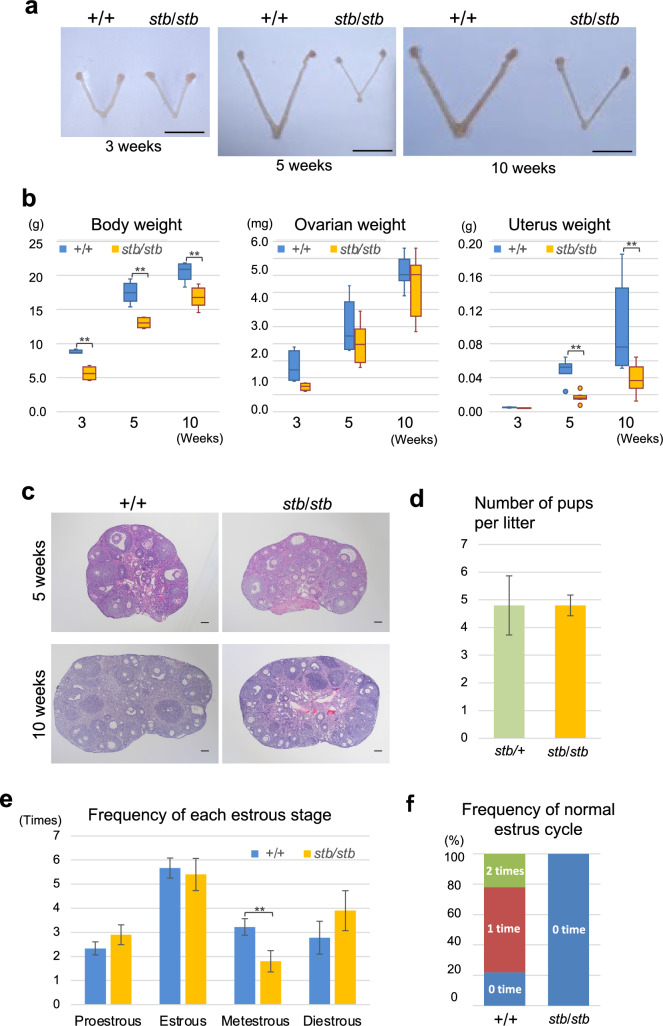
Analysis of the reproductive tract of *stb/stb* mice. **a** Comparison of the ovaries and uterus of +/+ and *stb*/*stb* littermates at 3, 5 and 10 weeks of age. The scale bar in each panel indicates 1.0 cm. **b** Comparison of the ovary and uterus weights of *stb*/*stb* and +/+ mice. The weights of the body and organs of *stb*/*stb* and +/+ mice were measured at 3 weeks (+/+ n = 3, *stb*/*stb* n = 3), 5 weeks (+/+ n = 8, *stb*/*stb* n = 8) and 10 weeks (+/+ n = 10, *stb*/*stb* n = 10) of age. 10-week-old mice were used at the proestrus stage, as determined by the vaginal smear test. Each box shows the 25–75% values. Bars denote minimum-maximum values, and the horizontal line in each box is the median. **c** Representative sections stained with hematoxylin and eosin of the ovaries of *stb*/*stb* and +/+ mice at 5 weeks and 10 weeks of age. The scale bar in each panel indicates 100 µm. **d** Comparison of litter sizes of female mice. At 11-12 weeks of age, female mice (*stb*/*stb* n = 4, *stb*/ + n = 4) were mated with *stb*/ + male mice. The number of pups at first parturition was counted. **e** Frequency of appearance of each estrous stage of *stb*/*stb* and +/+ mice. Vaginal smears of *stb*/*stb* (n = 10) and +/+ (n = 9) mice were collected daily for 14 days starting at 10 weeks of age. **f** Percentage of the number of normal estrous cycles in *stb*/*stb* (n = 10) and +/+ (n = 9) mice. The number of normal estrous cycles was determined based on the data used in (**e**). The normal estrous cycle was counted when proestrus, estrus, metestrus and diestrus were observed in order within 4–5 days. Data are presented as the mean ± SEM. **p < 0.01 indicates differences between *stb*/*stb* and +/+ mice

The testicular weights of *stb*/*stb* mice aged 30 and 60 days are markedly lower than those of control mice (Lane and Dickie 1968). Female *stb*/*stb* mice are fertile, although whether the reproductive tract is normal remains to be determined. We therefore observed the ovaries and uteri of *stb*/*stb* and +/+ mice at 3, 5, and 10 weeks of age. There were no clear differences in the external morphology of the ovaries at these times (Fig. 4a). Although the ovarian weights of the *stb*/*stb* mice tended to be lower than those of +/+ mice, the differences were not significant (Fig. 4b). Histological analysis of the ovaries revealed multiple normal follicles in each of the developmental stages from primordial to antral follicles, and no obvious differences were observed in *stb*/*stb* and +/+ mice at 5 and 10 weeks of age (Fig. 4c).

There was no clear difference in the appearances of the uteri between *stb*/*stb* and +/+ mice at 3 weeks of age. However, at 5 and 10 weeks of age, the uteri of *stb*/*stb* mice were markedly smaller than those of +/+ mice (Fig. 4a). The uterine weights of *stb*/*stb* mice at 3, 5, and 10 weeks of age were approximately 84%, 34%, and 40% of those of +/+ mice, respectively. The body weights of *stb*/*stb* mice at 3, 5, and 10 weeks of age were 64%, 75%, and 82% of those of +/+ mice, respectively, and a severe decrease disproportionate to decreases in body weight was observed in the uteri of *stb*/*stb* mice at 5 and 10 weeks of age (Fig. 4b). Despite severe uterine hypoplasia, *stb*/*stb* female mice have been reported to be fertile. However, it is unclear whether *stb*/*stb* female mice have a normal litter size. Therefore, we compared the number of pups at first parturition in *stb*/*stb* and normal (*stb*/+) female mice. As a result, no significant difference in litter size between *stb*/*stb* and *stb*/+ mice was observed (Fig. 4d).

Although there was no significant difference in litter size, we experienced that the reproductive efficiency of *stb*/*stb* female mice was not as high as that of *stb*/+ female mice. To analyze whether the cyclicity of the estrous cycle of *stb*/*stb* mice is normal, we examined the estrous cycle by checking vaginal smears of *stb*/*stb* and +/+ mice for 14 days from 10 weeks of age. The frequency of proestrus, estrus and diestrus stages was not significantly different between *stb*/*stb* and +/+ mice, whereas the frequency of metestrus was significantly lower in *stb*/*stb* mice than in +/+ mice (Fig. 4e). Furthermore, more than one normal estrous cycle consisting of proestrus, estrus, metestrus and diestrus for 4–5 days was observed in 80% of +/+ mice. In contrast, in *stb*/*stb* mice, the normal estrous cycle was not observed (Fig. 4f), and diestrus stages for more than 4 consecutive days were observed in 50% of *stb*/*stb* mice.

### Analysis of Adamtsl2 expression in the reproductive endocrine organs

**Fig. 5:**
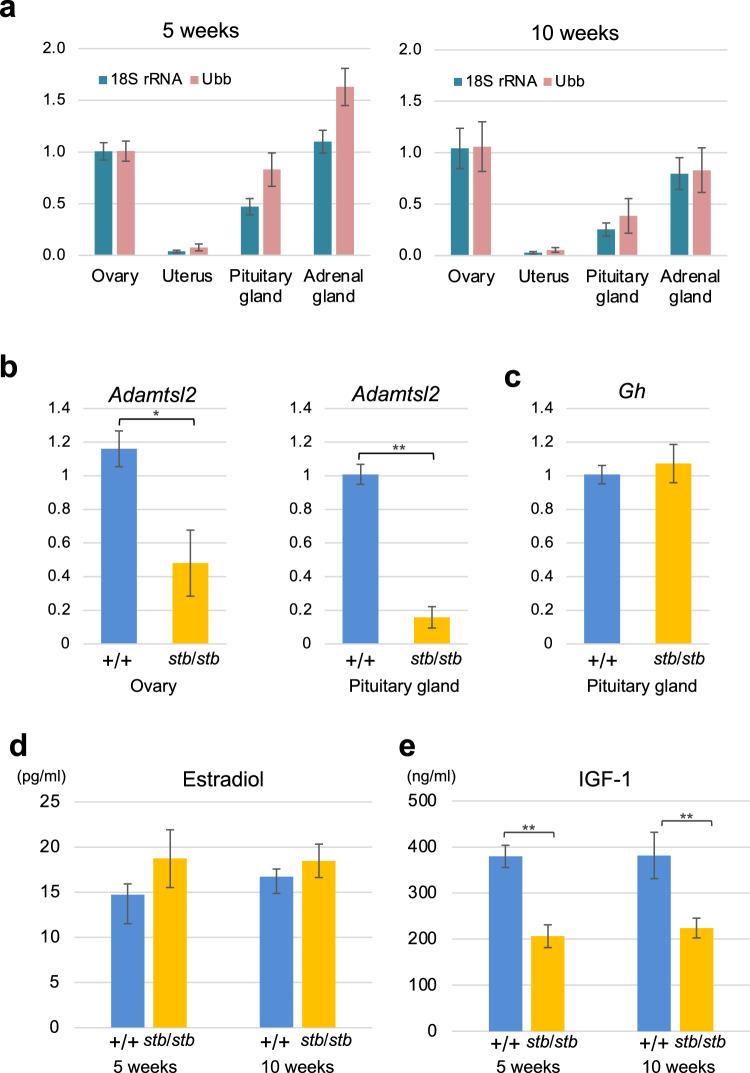
Analysis of *Adamtsl2* expression and endocrine factors in reproductive endocrine organs. **a** Expression levels of *Adamtsl2* in the ovaries, uterus, pituitary glands and adrenal glands of normal female mice. The expression levels of *Adamtsl2* were analyzed in +/+ mice at 5 weeks (n = 5) and 10 weeks (n = 5) of age. Mice at 10 weeks of age were used in the proestrus stage of the estrus cycle. For accurate data comparisons of gene expression levels between tissues, *18S rRNA* and *Ubb* genes were used as a reference for normalization. **b** Expression levels of *Adamtsl2* in the ovaries and pituitary glands of *stb*/*stb* and +/+ mice at 3 weeks of age (ovary: *stb*/*stb* n = 4, +/+ n = 4; pituitary glands: *stb*/*stb* n = 5, +/+ n = 5). **c** Expression levels of *Gh* in the pituitary glands of *stb*/*stb* and +/+ mice at 3 weeks of age (*stb*/*stb* n = 5, +/+ n = 5). **d** Plasma estradiol levels of *stb*/*stb* and +/+ mice at 5 weeks (+/+ n = 4, *stb*/*stb* n = 4) and 10 weeks (+/+ n = 4, *stb*/*stb* n = 5) of age. **e** Plasma IGF1 levels of *stb*/*stb* and +/+ mice at 5 weeks (+/+ n = 7, *stb*/*stb* n = 7) and 10 weeks (+/+ n = 7, *stb*/*stb* n = 7) of age. Data are presented as the mean ± SEM. *p < 0.05, **p < 0.01 indicates differences between *stb*/*stb* and +/+ mice

According to mouse ENCODE transcriptome data on NCBI, in adult mice, *Adamtsl2* expression is highest in the lungs, followed by the ovaries and adrenal glands. However, information regarding the expression levels in the uterus and all other organs in younger mice is not available. We therefore analyzed the expression levels of *Adamtsl2* in the ovary, uterus, pituitary glands and adrenal glands of normal (+/+) mice at 5 and 10 weeks of age. For more accurate data comparisons of gene expression levels between tissues, *18S rRNA* and *Ubb* genes were used as a reference for normalization in quantitative real-time PCR. The expression levels in the ovary and adrenal gland were similar at 5 and 10 weeks of age, while the expression levels in the uterus were approximately 6% and 4% of those in the ovary at these times (Fig. 5a). The expression levels in the pituitary glands were approximately 65% of the levels in the ovaries at 5 weeks (Fig. 5a). Furthermore, we analyzed the expression levels of *Adamtsl2* in the ovaries and pituitary glands of *stb*/*stb* and +/+ mice at 3 weeks of age. The results showed that the expression levels of *Adamtsl2* in the ovaries and pituitary of *stb*/*stb* mice were reduced to approximately 41% and 16% of normal mice, respectively (Fig. 5b). These decreased levels of *Adamtsl2* mRNA, which contains a premature termination codon due to the nonsense mutation, are expected to be due to degradation by nonsense-mediated mRNA decay.

### Analysis of GH, estradiol and IGF1 levels in *stb/stb* mice

From 3 to 5 weeks of age, when uterine hypoplasia and growth retardation occur in *stb*/*stb* mice, endocrine factors produced by the pituitary glands and ovaries strongly influence uterine development and bone growth. This study showed that the expression level of *Adamtsl2* in normal mice was very low in the uterus, while it was high in the ovary and pituitary gland. These expression levels were markedly reduced in *stb*/*stb* mice. We therefore hypothesized that the abnormalities in *stb*/*stb* mice were caused by decreased levels of endocrine factors derived from the ovaries, pituitary glands, or both. Thus, we analyzed the expression levels of growth hormone (*Gh*) in the pituitary gland and the concentrations of plasma estradiol and IGF1, which are important endocrine factors for uterine development and bone growth. The results showed that no significant differences in *Gh* expression levels (Fig. 5c) and plasma estradiol levels (Fig. 5d) were observed between *stb*/*stb* and +/+ mice. In contrast, the plasma IGF1 levels of *stb*/*stb* mice at 5 and 10 weeks of age were significantly lower than those of +/+ mice (54% and 59% of the IGF1 concentrations of +/+ mice at 5 and 10 weeks of age, respectively) (Fig. 5e).

## Discussion

In the present study, we identified a nonsense mutation in *Adamtsl2* as the cause of the phenotype of *stb*/*stb* mice. Furthermore, hypoplasia of the uterus and an irregular estrous cycle are newly discovered abnormalities in *stb*/*stb* mice. Therefore, together with the known infertility of *stb*/*stb* male mice (Chubb and Nolan 1985; Chubb and Henry 1987), our present results revealed that *Adamtsl2* contributes to the regulation of male and female reproductive function and development.

Here, we showed that *stb*/*stb* mice exhibited dwarfism with shortened longitudinal bones and thickened skin, which are consistent with the abnormalities caused by mutated *ADSMTSL2*/*Adamtsl2* genes in humans (Le Goff et al. 2008; Piccolo et al. 2019; Allali et al. 2011; Steinle et al. 2021), dogs (Bader et al. 2010) and mice (Hubmacher et al. 2015). In addition, the present histological analysis of the tibial growth plate revealed that *stb*/*stb* mice showed a remarkable reduction in the hypertrophic chondrocyte layer. This indicates that the shorter longitudinal bones of *stb*/*stb* mice are caused by impaired endochondral ossification, similar to *Adamtsl2*-KO mice (Delhon et al. 2019). Thus, we conclude that the nonsense mutation in *Adamtsl2* causes phenotypic abnormalities in *stb*/*stb* mice.

Each member of the ADAMTS family possesses a specific domain structure within the C-terminal ancillary domain, which is predicted to confer functional specificity. The nonsense mutation that we identified in *Adamtsl2* was within codon 747 in the fifth thrombospondin type 1 domain. Thus, although we confirmed that *Adamtsl2* mRNA levels were reduced by more than 50% in *stb/stb* mice, *stb*/*stb* mice might generate a truncated ADAMTSL2 that lacks the fifth to seventh thrombospondin type 1 domains and PLAC module from undegraded *Adamtsl2* mRNA. Missense mutations have been reported within the C-terminal ancillary domain of ADAMTSL2 in patients with GPHYSD1 (Le Goff et al. 2008; Allali et al. 2011). Some types of these mutations cause reduced secretion of ADAMTSL2 by loss of *O*-fucosylation on thrombospondin type 1 domains (Zhang et al. 2020). These findings indicate that thrombospondin type 1 domains are essential for ADAMTSL2 to function normally.

*Adamtsl2*-conditional KO mice exhibit dwarfism immediately after birth, which is accompanied by a narrow hypertrophic chondrocyte layer of the growth plate and disrupted column formation of chondrocytes until at least 30 days postpartum (Delhon et al. 2019). However, *stb*/*stb* mice exhibit dwarfism from 1 week of age, and the decrease in the hypertrophic chondrocyte layer observed in *stb*/*stb* mice at 3 weeks of age was difficult to detect at 5 weeks of age. Moreover, *Adamtsl2*-KO mice die at birth from severe bronchial epithelial dysplasia (Hubmacher et al. 2015). In contrast, *stb*/*stb* mice rarely immediately die after birth and normally mature without suffering from detectable lung abnormalities. These observations indicate that the disorders in *stb*/*stb* mice are milder than those in *Adamtsl2*-KO mice. This may be explained by the presence of a truncated ADAMTSL2 that retains some normal function.

Numerous studies have shown that the members of the ADAMTS superfamily, comprising 19 secreted ADAMTS and 7 secreted ADAMTSL in mammals, contribute to reproductive functions. In particular, ADAMTS is better characterized than ADAMTSL. Several ADAMTS proteases are expressed in male and female reproductive organs, and some genes encoding ADAMTS proteases are associated with reproductive disorders (Russell et al. 2015). Male *Adamts2*-KO and *Adamts*16-KO mice are infertile and are associated with a marked decrease in testicular sperm and cryptorchidism, respectively (Li et al. 2001; Abdul-Majeed et al. 2014). *Adamts1*-KO mice are subfertile with impaired ovulation (Shindo et al. 2000; Mittaz et al. 2004). Polymorphisms in human *ADAMTS19* are associated with premature ovarian failure (Pyun et al. 2015). Thus, ADAMTS proteases contribute diverse and important functions to male and female reproduction, and understanding the roles of ADAMTS proteases is expected to offer therapeutic targets to resolve infertility, biomarkers that predict dysfunction of the reproductive organs, and targets for development of nonhormonal male and female contraceptives (Russell et al. 2015). However, there have been no reports directly demonstrating the importance of ADAMTSL, including ADAMTSL2, in mammalian reproductive function.

Previous studies have shown that *stb*/*stb* male mice have abnormally small testes from 29 to 32 days of age and infertility due to impotence (Lane and Dickie 1968; Chubb and Nola 1985; Chubb 1987). while *stb*/*stb* female mice are reported to be able to produce offspring (Lane and Dickie 1968). In addition, this study showed no difference in the number of pups at first parturition between *stb*/*stb* and normal (*stb*/+) female mice at 11–12 weeks of age. However, the present study revealed that uterus development and the estrous cycle are abnormal in *stb*/*stb* female mice. We additionally observed more severe uterine hypoplasia in *stb*/*stb* mice at 5 weeks of age than in those at 3 weeks of age. These observations suggest that *Adamtsl2* plays an important role not only in bone growth but also in the development of the uterus and testes during the pubertal period in mice. Intriguingly, despite the severe uterine hypoplasia at 5 weeks of age, *stb*/*stb* female mice at 11–12 weeks of age can produce offspring. One possible explanation could be the potential recovery or compensatory growth. This seems to be supported by the diminishing difference in uterine weight (34% and 40% at 5 and10 weeks of age, respectively) between *stb*/*stb* and +/+ mice. This progressive normalization of uterine size might play a crucial role in the fertility of *stb*/*stb* mice even though the uterus doesn't achieve the size found in +/+ mice.

Several studies have shown that ADAMTSL2 binds FBN1 and latent TGFβ-binding protein-1 to modulate the activities of TGFβ signaling pathways (Le Goff et al. 2011; Sengle et al. 2012). Increased activation of TGFβ1 signaling occurs in humans with GPHYSD1 (Le Goff et al. 2008; Piccolo et al. 2019), dogs with Musladin Lueke Syndrome (Packer et al. 2017) and *Adamtsl2*-KO mice (Hubmacher et al. 2015), and evidence indicates that these alterations are the most likely cause of the abnormalities as a consequence of impaired ADAMTSL2. However, treatment with a TGFβ-neutralizing antibody does not correct bronchial epithelial dysplasia in *Adamtsl2*-KO mice (Hubmacher et al. 2015), implying that other factors mediate ADAMTSL2 function. Furthermore, the uteri of mice with a constitutively activated TGFβ1 receptor are enlarged (Gao et al. 2015), exhibiting the exact opposite response to hypoplasia of the uterus of *stb*/*stb* mice. Thus, we believe that it is reasonable to consider that *Adamtsl2* may be involved in a regulatory mechanism employed by the reproductive system that is not mediated by TGFβ signaling.

Depletion of growth hormone disrupts the estrous cycle in female mice (Bartke 1999; Hull and Harvey 2001), and these mice exhibit a smaller body size (List et al. 2019; Zhou et al. 1997). These abnormalities represent the phenotype of *stb*/*stb* mice. Moreover, our results showed that the expression levels of *Adamtsl2* were higher in the pituitary gland of normal mice, and we assumed that *Adamtsl2* is involved in the regulation of growth hormone expression in the pituitary gland. However, *Gh* expression levels in the pituitary gland of *stb*/*stb* mice were not reduced. In contrast, we found a 54–59% reduction in plasma IGF1 levels in *stb*/*stb* mice, although IGF1 levels in the blood depend on growth hormone levels produced by the pituitary gland (Sjögren et al. 1999; Yakar et al. 1999). Since uterine hypoplasia occurs in IGF1-KO mice (Baker et al. 1996), the uterine hypoplasia of *stb*/*stb* mice may be explained by reduced IGF1 levels. Furthermore, *stb*/*stb* male mice are a mouse model for infertility due to impotence (Chubb and Henry 1987). Previous reports suggest that the growth hormone-IGF1 axis is involved in normal penile development (Laron and Sarel 1970; Laron and Klinger 1998; Cannarella et al. 2021) and impotence (Fujita et al. 1997). Although further studies are needed to determine the extent to which reduced levels of IGF1 are implicated in the abnormalities observed in *stb*/*stb* mice, the understanding of the relationship between *Adamtsl2* and IGF1 might provide important insights into the regulation of female reproductive function and development, as well as male infertility caused by impotence.

Our findings regarding the involvement of *Adamtsl2* in the regulation of uterine development and the sexual cycle in females indicate a new physiological role of the members of the ADAMTS family. However, interacting factors and signaling pathways involved in ADAMTSL2 in the extracellular matrix of reproductive organs remain to be identified, and a better understanding of the functions of ADAMTSL2 will likely reveal a novel regulatory mechanism of the reproductive system mediated by the extracellular matrix.

## Data Availability

The nucleotide sequence data from this study have been submitted to the DDBJ Sequence Read Archive under accession number DRA014778. The datasets generated and/or analyzed during the current study are available from the corresponding author on reasonable request.
